# Structure of a helicase–helicase loader complex reveals insights into the mechanism of bacterial primosome assembly

**DOI:** 10.1038/ncomms3495

**Published:** 2013-09-19

**Authors:** Bin Liu, William K. Eliason, Thomas A. Steitz

**Affiliations:** 1 Department of Molecular Biophysics and Biochemistry, Yale University, New Haven, Connecticut 06520, USA; 2 Howard Hughes Medical Institute, New Haven, Connecticut 06510, USA; 3 Department of Chemistry, Yale University, New Haven, Connecticut 06520, USA

## Abstract

During the assembly of the bacterial loader-dependent primosome, helicase loader proteins bind to the hexameric helicase ring, deliver it onto the *ori*C DNA and then dissociate from the complex. Here, to provide a better understanding of this key process, we report the crystal structure of the ~570-kDa prepriming complex between the *Bacillus subtilis* loader protein and the *Bacillus stearothermophilus* helicase, as well as the helicase-binding domain of primase with a molar ratio of 6:6:3 at 7.5 Å resolution. The overall architecture of the complex exhibits a three-layered ring conformation. Moreover, the structure combined with the proposed model suggests that the shift from the ‘open-ring’ to the ‘open-spiral’ and then the ‘closed-spiral’ state of the helicase ring due to the binding of single-stranded DNA may be the cause of the loader release.

## Abstract (web summary)

During the initiation of bacterial DNA replication, loader proteins transfer the hexameric helicase ring onto replication origin DNA. Liu *et al.* report the crystal structure of a 570-kDa prepriming complex and suggest that the release of loader proteins is associated with the transition of the helicase ring to a spiral configuration.

## Graphical abstract

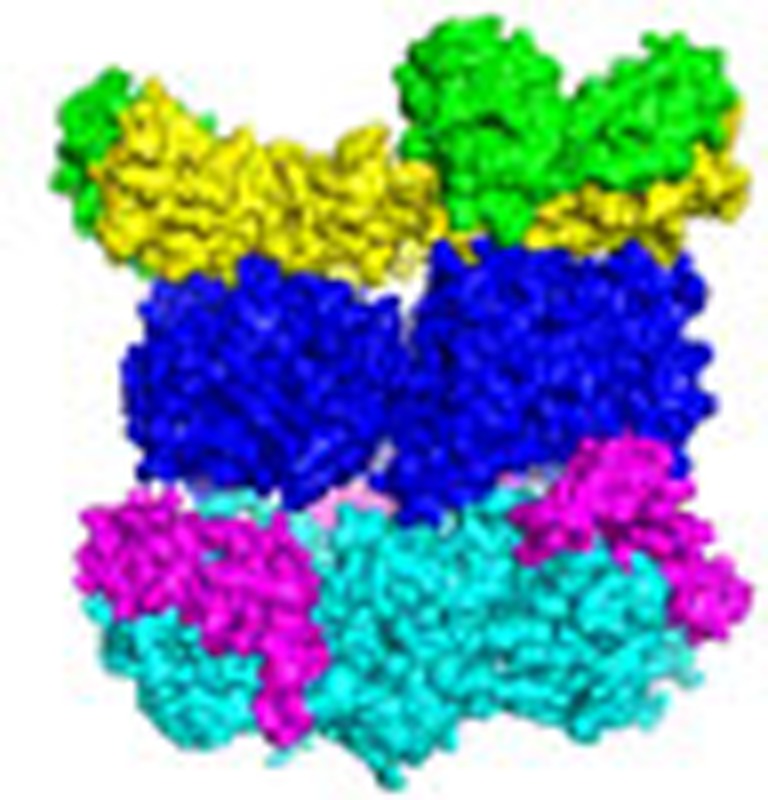

The replication of the bacterial chromosome is initiated at *ori*C where the initiator protein DnaA binds to start the assembly of the enzymatic replisome machine^1^. The early stages of this process involve the assembly of the primosome and the formation of a functional primosome^2^,^3^. Subsequent to the remodelling of the replication origin induced by DnaA, the assembly of the bacterial loader-dependent primosome occurs in discrete steps and involves at least four different proteins (DnaA, helicase, helicase loader and primase) that act in a coordinated and sequential manner.

In the *Escherichia coli* system, the helicase loader protein, DnaC, complexed with ATP, binds to hexameric helicase DnaB and forms a DnaB_6_–DnaC_6_ complex, which has been confirmed by cryo-electron microscope (cryo-EM) studies^4^,^5^. The loader protein delivers the helicase onto the melted DNA single strands of the DnaA–*ori*C nucleoprotein complex at the origin of replication^2^,^6^. *In vivo*, this delivery is associated with the initiator protein DnaA^2^,^7^, whose amino-terminal domain (NTD) is thought to have a role in loading the helicase and helicase loader complex onto the *ori*C by interacting with helicase DnaB^8^. After the loader protein dissociates from the helicase ring, the NTD of the helicase interacts with the carboxy-terminal domain (CTD) of the primase and forms a functional primosome that synthesizes RNA primers^9^. Primosome assembly in Gram-positive bacteria is different in the details, including that the corresponding helicase is named DnaC in some bacteria such as *Bacillus subtilis*, and the loader protein is DnaI; the assembly of the helicase and loader protein complex onto the replication origin is assisted by a pair of co-loader proteins DnaB and DnaD in *B. subtilis*^10^,^11^,^12^,^13^.

The hexameric helicase DnaC in *B. subtilis* is inactive in the unwinding activity^14^ and less stable than the *E. coli* DnaB, and can easily form a mixture of different oligomeric states^12^. However, the stable hexameric DnaB helicase in *B. stearothermophilus* (BstDnaB) was observed to form a quite stable complex with the loader protein DnaI in *B. subtilis* (BsuDnaI)^15^,^16^. The replicative hexameric DnaB helicase is composed of two domains that are connected by one flexible linker^17^,^18^. The larger CTD exhibits a classical RecA fold, binds the nucleoside triphosphate and translocates the protein on the DNA, whereas the NTD surrounds the single-stranded DNA (ssDNA) during DNA unwinding and binds the primase^19^,^20^. DnaB helicase can bind to ssDNA but not to the *ori*C in the absence of loader protein^3^,^21^. The crystal structures of various hexameric helicases in complex with ssDNA or single-stranded RNA show a spiral staircase of ssDNA or single-stranded RNA passing through the central channel of the helicase ring^22^,^23^,^24^.

The DnaI helicase loader belongs to the AAA+ family of ATPases^25^,^26^. The CTD consists of an AAA+ fold that is required for nucleotide and ssDNA binding. Its smaller NTD functions as a molecular switch to regulate the binding of DNA^16^. The NTD of this DnaI loader, which contains a novel zinc-binding fold, is not homologous to that of *E. coli* loader protein DnaC^27^, and was found to interact with the CTD of the DnaB helicase in the complex of DnaB with DnaI^15^. The loading of the hexameric helicase ring onto ssDNA can be achieved by the helicase loader or the primase-like domain of the helicase^28^. Two different mechanisms of the loader-dependent loading have been proposed, based on biochemical studies: helicase ring-breaking and ring-making^12^,^29^,^30^. In some species, such as *E. coli*, helicase loader is believed to aid the parting of a subunit interface in the stable helicase ring and thereby break the ring. However, in other species, such as *B. subtilis*, the oligomeric helicases can be assembled into a hexamer ring around DNA by the helicase loader, which acts as a ring maker. There are two reported cryo-EM structures of the *E. coli* helicase–helicase loader complex that exhibit different quaternary structures^4^,^5^. The more recent cryo-EM structure shows a spiral arrangement of the two interacting hexamers with a split ring, consistent with the possibility of a ring-breaking role of the loader^5^.

The translocation of the helicase ring in a 5′–3′ direction on the *ori*C transports primase to the initiation sites and enables the synthesis of RNA primers. Previous studies using gel filtration, fluorescence, cross-linking and atomic force microscopy indicated that primase DnaG binds to helicase to form a functional primosome with variable stoichiometries that vary from one to three primase molecules to one hexameric helicase ring^19^,^31^,^32^. However, our recent structure of BstDnaB in complex with GDP and ssDNA substrate suggests that only one primase is included in the complex when it functions in making RNA primers^24^. The DnaG primase consists of three domains: an N-terminal zinc-binding domain, an RNA polymerase domain and a C-terminal helicase-binding domain (HBD)^33^. Functional interactions between DnaB helicase and DnaG primase show that DnaG stimulates the ATPase and the helicase activities of DnaB^19^, and DnaB modulates and increases the synthesis of RNA primers^34^. It has been believed that loader protein would be ejected from the helicase and loader complex mounted on the *ori*C before primase binds to the NTD of helicase. However, recent studies that show the existence of a stable complex between helicase, loader protein and primase^15^, the synthesis of RNA primers in the presence of the loader protein *in vitro*^14^,^31^ and the effects of ribonucleotides and primase on the release of the loader protein from the helicase and loader complex^35^ have suggested that the release of loader protein is coupled with the movement of the helicase ring and the synthesis of the RNA primer, and not the initial recruitment of primase.

Although biochemical studies have shed light on the mechanisms of primosome assembly, crystal structures of the complexes that occur during primosome assembly are quite limited. Here we report a crystal structure of BstDnaB helicase in complex with BsuDnaI loader protein and the HBD of BstDnaG primase at 7.5 Å resolution. This structure shows how the DnaB helicase interacts with the DnaI loader with a stoichiometry of one hexameric helicase ring binding to three loader protein dimers and three HBDs of primase DnaG. Moreover, the structure and the proposed model suggest that the release of helicase loaders may result from the binding of ssDNA to the helicase ring, which is accompanied by the shift from the ‘open-ring’ to the ‘open-spiral’, and then the ‘closed-spiral’ state of the helicase ring.

## Results

### Structure determination

**Figure 1:**
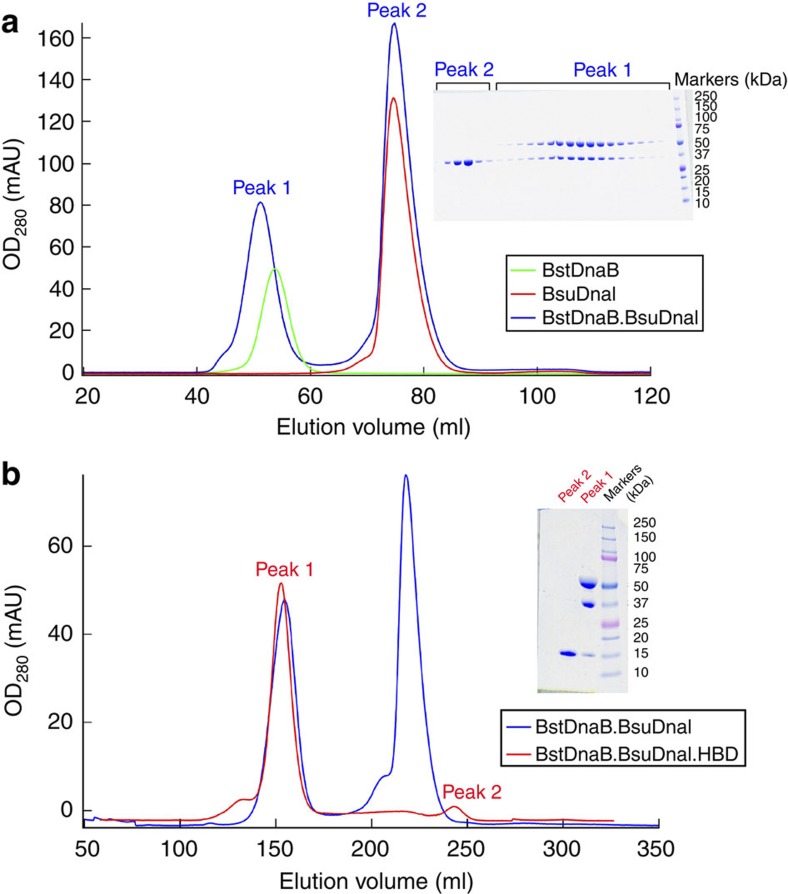
The interactions of BstDnaB, BsuDnaI and the HBD of BstDnaG examined by size-exclusion chromatography. (**a**) Green, red and blue traces represent the migrations of BstDnaB, BsuDnaI and a premixture of BstDnaB and extra BsuDnaI, respectively, on a 16/60 Superdex G200 prep grade gel filtration column with a flow rate of 1 ml min^−1^. The inserted SDS–PAGE gel verifies the presence of the BstDnaB–BsuDnaI complex in peak 1. (**b**) Blue and red curves show the migrations of a premixture of BstDnaB and extra BsuDnaI and a premixture of the BstDnaB–BsuDnaI complex and extra HBD of BstDnaG, respectively, on a 26/60 Superdex G200 prep grade gel filtration column with a flow rate of 1 ml min^−1^. The corresponding peak 1 on the gel indicates the formation of a stable BstDnaB–BsuDnaI–HBD complex. The figure was created using Igor Pro.

The complex of BstDnaB helicase, BsuDnaI loader and the HBD of BstDnaG primase was obtained by first purifying the DnaB–DnaI complex using gel filtration chromatography and then adding the HBD of DnaG (Fig. 1). This procedure is the same as previously reported^15^. Analysis of the proteins in these crystals using mass spectrometry and tryptic digestion confirmed the presence of these three different proteins. In addition, another crystal form was also obtained under different conditions and was shown to contain one hexameric DnaB and three HBDs of DnaG by using molecular replacement. Further, calculation of the Matthews coefficient confirmed the existence of hexameric DnaB, hexameric DnaI and three HBDs of DnaG in an asymmetric unit.

**Figure 2:**
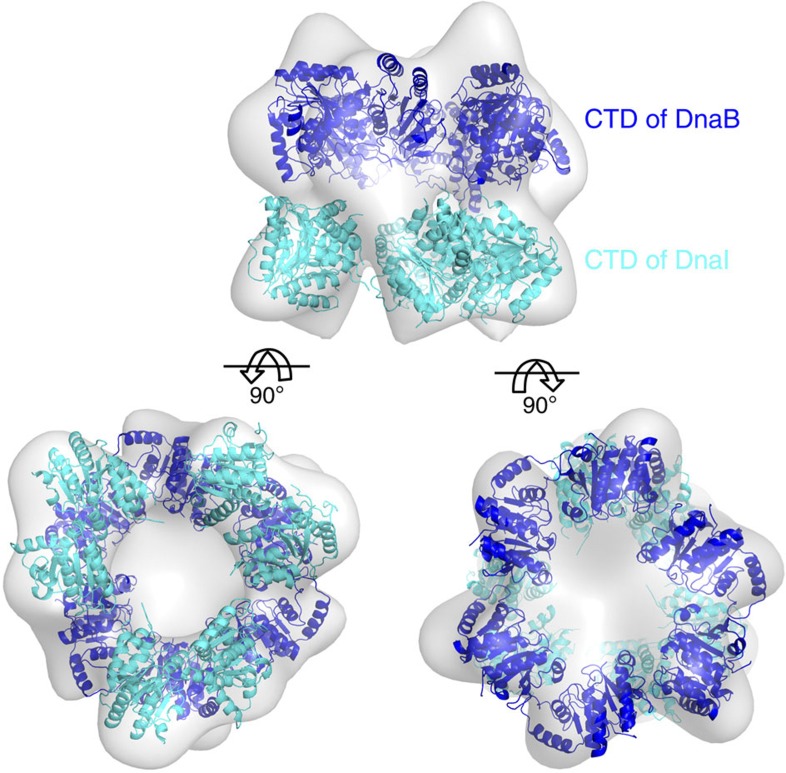
Fitting of the CTDs of both BstDnaB and BsuDnaI into the cryo-EM map of the *E. coli* helicase and helicase loader complex. The CTDs of BstDnaB and BsuDnaI are shown in blue and cyan, respectively. The grey surface of the cryo-EM density map is contoured at 5 σ.

The structure was determined by molecular replacement. Initially, subunits or single proteins were used as search models. We then built a new model containing as many components as possible that were positioned using the cryo-EM density map of the *E. coli* helicase and helicase loader complex^4^ (EMD-1017, EMDB accession code). The BstDnaB helicase and the CTD of BsuDnaI are homologous to the helicase and the CTD of helicase loader in *E. coli*, respectively. However, it was believed that there is a structural difference between the NTD of BsuDnaI and the NTD of *E. coli* DnaC^27^. Therefore, a model containing the CTDs of BstDnaB and BsuDnaI was positioned into the cryo-EM density map (Fig. 2). As the structures of the different crystals containing DnaB and the HBDs of DnaG mentioned above are quite similar to the structure of BstDnaB in complex with the HBD of BstDnaG (2R6C, PDB accession code), we assumed the conformation of the NTDs of DnaB and the HBDs of DnaG should be the same as the 2R6C structure^18^. Consequently, the NTDs of BstDnaB and the HBDs of BstDnaG were added into the model by superimposing the CTDs of the helicase in the model on those of the BstDnaB and HBD complex structure (2R6C), which generated a new model. The averaged maps (Supplementary Fig. S1a,b) created by refining and averaging BstDnaB alone without including the HBDs or the loaders from the beginning provide support for the model.

**Figure 3:**
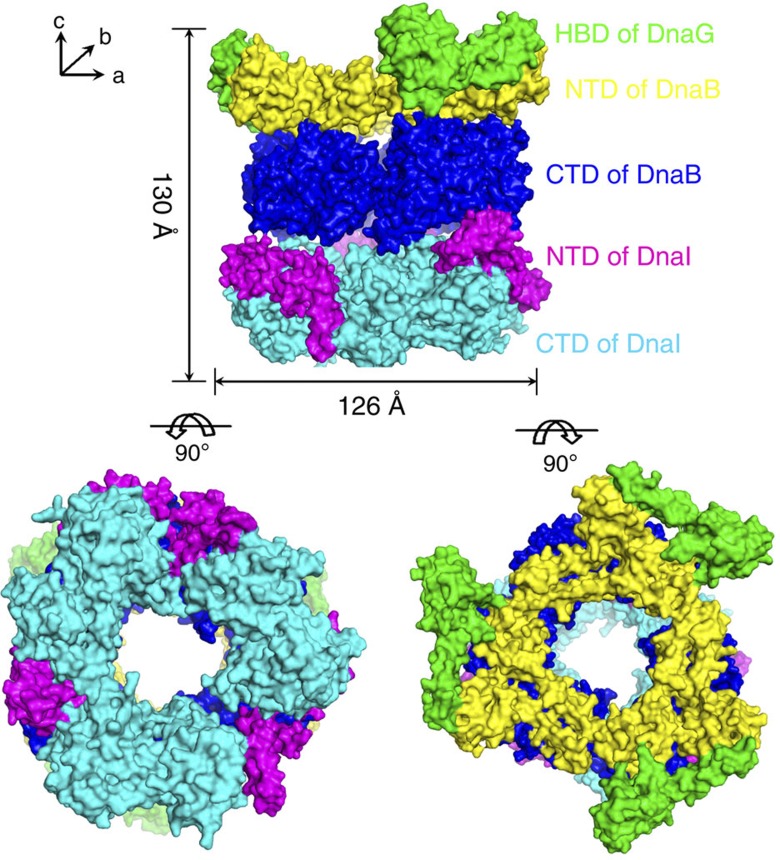
Views of the overall structure of BstDnaB in complex with BsuDnaI and HBD of BstDnaG. The surface representations of the structure of the complex containing the HBDs of BstDnaG (green), the NTDs of BstDnaB (yellow), the CTDs of BstDnaB (blue), the NTDs of BsuDnaI (magenta) and the CTDs of BsuDnaI (cyan) are shown along the *c* and *a* axes of crystal lattice. The lengths of the complex along these axes are around 130 Å and 126 Å, respectively.

**Figure 4:**
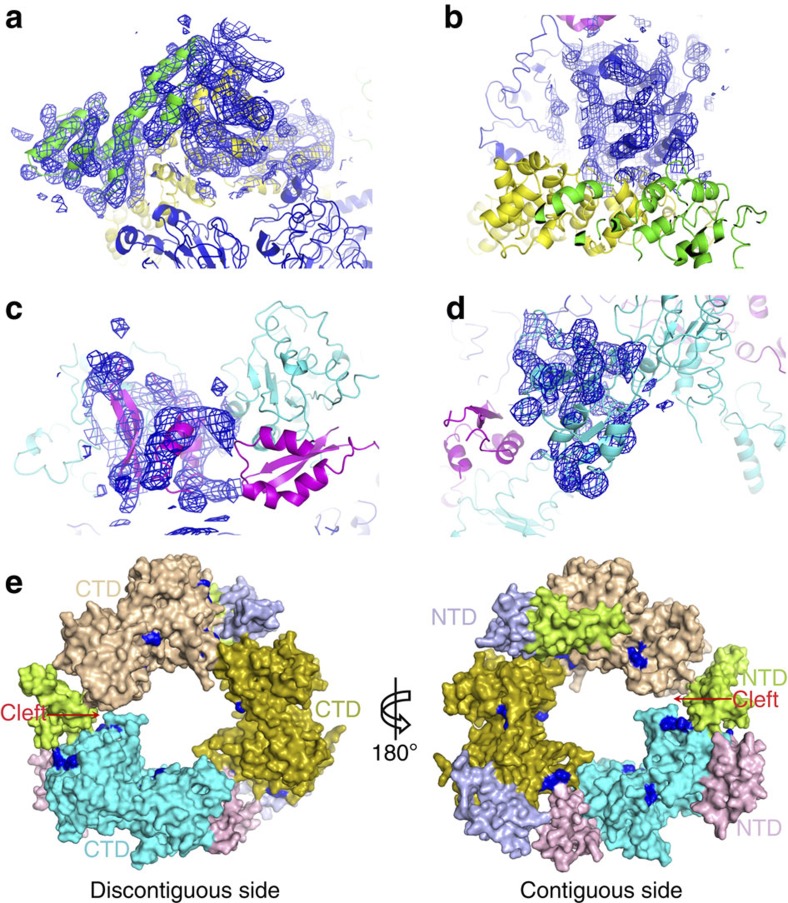
Views of the averaged electron density maps in different parts of the complex and the rings of the helicase loader. The colour scheme (**a**–**d**) for the proteins is same as in Fig. 3. (**a**) The blue averaged map, contoured in at 1.5 σ, covers one HBD of BstDnaG (chain I) and one NTD of BstDnaB (chain E). (**b**) The region close to the RecA fold in chain A of BstDnaB is superimposed on a 1.0 σ averaged map in blue. (**c**) One magenta NTD of BsuDnaI (chain K) is superimposed on the blue map contoured at 1.0 σ. (**d**) The blue averaged map contoured at 1.5 σ is superimposed on one AAA+ fold region of BsuDnaI (chain O). (**e**) Surface representations of the hexameric loader ring as seen in the prepriming complex structure viewed along the *c* axis of crystal lattice. The CTDs of the three dimers are cyan, olive and wheat, respectively. The corresponding NTDs are labelled as light pink, light blue and pale green, respectively. The conserved potential DNA-binding sites (Lys160, Lys162, Lys286, Arg289 and Arg293) are labelled with dark blue. The left figure is discontiguous side of the ring. The right figure is the contiguous side making contacts with the CTDs of the helicase ring. The observed cleft in the CTD ring of the loaders is labelled with red.

Initial phases were obtained using the new model that was created using the cryo-EM density as a guide. The phases calculated from the model were improved through threefold or sixfold non-crystallographic symmetry averaging of the different proteins or domains. Because of the modest resolution of the X-ray data, coordinates were optimized by the adjustment of the positions of the α-helices using the averaged maps and rigid-body refinements. Although the side chains are not observable at this resolution, they are included in the refinements, as they greatly improve the *R*_free_ value. The NTDs of BsuDnaI were manually fitted into averaged maps and 2*F*_o_–*F*_c_ density maps generated using the phases that were improved by symmetric averaging. The final coordinates of the whole model produce an *R*_factor_ of 0.379 and an *R*_free_ of 0.392 (Fig. 3 and Supplementary Table S1). The electron density map clearly shows the α-helices as rods at the current resolution (Fig. 4a–d). The 2*F*_o_–*F*_c_ omit map that was calculated after omitting the HBDs of BstDnaG together with the NTDs of BstDnaB in the complex provides support to the phasing obtained by molecular replacement (Supplementary Fig. S1c). The real-space correlation coefficients for fitting five regions of the model into the maps are shown in Supplementary Table S2.

### Overall structure and interactions

The oligomeric protein complex whose structure we determined contains two hexameric proteins, the helicase and the helicase loader, as well as three HBDs of the primase that are all required in bacterial primosome assembly. The protein stoichiometry in this structure is consistent with that of previously reported structures^4^,^18^ or biochemical studies, including gel filtration analysis^12^, co-expression and fusion protein expression of the helicase and the helicase loader^36^, surface plasmon resonance experiments using a Biacore^16^ and fluorescence measurements^37^.

The overall architecture of the 570-kDa complex exhibits a three-layered ring structure along the *c* axis of the crystal (Fig. 3). The NTDs of BstDnaB together with the HBDs of BstDnaG form the first ring that lies on the top of the second ring, which consists of the CTDs of BstDnaB. This double-layered ring structure resembles our previous structure of the helicases with HBDs (PDB accession code: 2R6C) with an root mean square deviation of 2.44 Å between corresponding Cα atoms. The NTDs of BstDnaB pack into a more rigid triangular collar shape that exhibits a threefold symmetry, whereas the CTDs have a relatively loose collar shape with a pseudo sixfold symmetry. The BsuDnaI on the bottom forms the loosest triangular collar of trimer of dimers with an obvious threefold symmetry as well (Fig. 4e). The complex assembly has an outer diameter of 145 Å at the site of the HBDs of DnaG, 120–126 Å at the other domains and a height of 130 Å. The diameter of the central channel is around 50–55 Å throughout its length, which is wide enough to allow either double-stranded DNA or ssDNA to pass through the channel. The Rho helicase complexed with nucleotide and RNA exhibited a ‘closed’ state at the CTDs of the helicase ring rather than the ‘open’ state of the apo-Rho helicase^23^. The diameter of the inner channel formed by the CTDs of BstDnaB is significantly smaller in the presence of substrates, as is also the case with DnaB complexed with DNA and nucleotide^24^.

The architecture of the complex is maintained by the interactions among its component proteins. The NTDs of the hexameric BstDnaB helicase interact with all the HBDs of primase BstDnaG using a contact surface area of ~2,756 Å^2^, resulting in a tight interaction. The ring of the CTDs of the helicase was previously reported to be held together primarily through their interactions with the linker regions of the helicase^18^. In this complex, it was also observed that the mainly hydrophobic interactions between the CTDs and the NTDs of the helicase bury a total contact surface area of ~2,415 Å^2^ per hexamer, which contributes the ring packing of the CTDs as well. The CTDs of hexameric BstDnaB helicase interact with both the NTDs and the CTDs of the helicase loader BsuDnaI with a total contact surface area of ~1,745 Å^2^ per hexamer. As the total contact surface among the three dimers of the helicase loader is ~1,502 Å^2^, the packing of the loader ring is probably maintained by both the interactions between domains and the contacts with the CTDs of the helicase.

The structure of this complex shows that the CTDs of the helicase loader interact directly with the helicase ring, which may have important mechanistic implications. This observation is consistent with the results from previous yeast two-hybrid experiments, which indicated weak interactions between the CTDs of BsuDnaI and BstDnaB helicase^16^. In this structure, the CTDs of BsuDnaI interact with the CTDs of BstDnaB in the vicinity of residues 211–212 of one monomer and around residues 281–282 of the other monomer in each dimer. These residues are not located in the conserved nucleotide-binding sites. It is interesting that not all the CTDs of the helicase loader make contact with the helicase. The CTDs of one dimer (chains N and O) exhibit a different orientation from the others and do not directly interact with the CTDs of the helicase. The contact surface area between the NTDs of BsuDnaI and the CTDs of BstDnaB is around 1,197 Å^2^, but that between the CTDs of BsuDnaI and BstDnaB is only ~548 Å^2^. Therefore, it appears that the NTDs of BsuDnaI have the major role in recruiting the helicase loader into the prepriming complex.

### The loader protein DnaI

Our analysis by gel filtration and multiple-angle light scattering (Supplementary Fig. S2a) has demonstrated that the helicase loader is a mixture of dimers and monomers with a preference for the monomer (~93% monomer of all samples at a concentration of 10 mg ml^−1^) in solution. This result differs from the previous reports on the overexpression of helicase loaders that concluded it was a monomer in solution^12^,^25^,^36^; however, it apparently provides support for the observation that the helicase loader dimers bind to the helicase ring.

The three dimers of the helicase loader form a ring that exhibits threefold symmetry and contacts the CTDs of the helicase with both their CTDs and NTDs through hydrophobic and polar interactions. The different orientation of the CTDs of one dimer (chain N and O) compared with the other two results in a slightly spiral CTD ring and an obvious cleft that appears in the contact region (Fig. 4e). In addition, as shown in Fig. 4e, the previously reported conserved potential DNA-binding residues (Lys160, Lys162, Lys286, Arg289 and Arg293) on the CTDs of the helicase loader^36^ are observed in two different orientations. The residues on one monomer of the loader dimer face towards the NTD of the molecule it contacts. These residues might function together with the nearby NTD in the initial step of binding DNA. Those on the other monomer face towards the inner channel of the loader ring and probably have roles in further stabilizing and holding DNA.

The cryo-EM study of Barcena *et al.*^4^ determined that the *E. coli* helicase loader DnaC interacts asymmetrically with the helicase DnaB. The loader DnaC makes extensive contacts with the hexameric DnaB ring and exhibits a relative twist angle of ~50° among DnaC and DnaB dimers. In the present crystal structure, the two monomers in each dimer of the helicase loader make distinct interactions with the helicase. One monomer contacts two CTDs of the helicase, whereas the other one interacts with only one CTD of the helicase. These observations are similar to the conclusions drawn from the cryo-EM study. In addition, the two monomers of each dimer of this loader DnaI do not exhibit twofold symmetry relative to each other, as the positions of their NTDs relative to their CTDs differ.

### The ‘spiral’ states of the helicase ring

**Figure 5:**
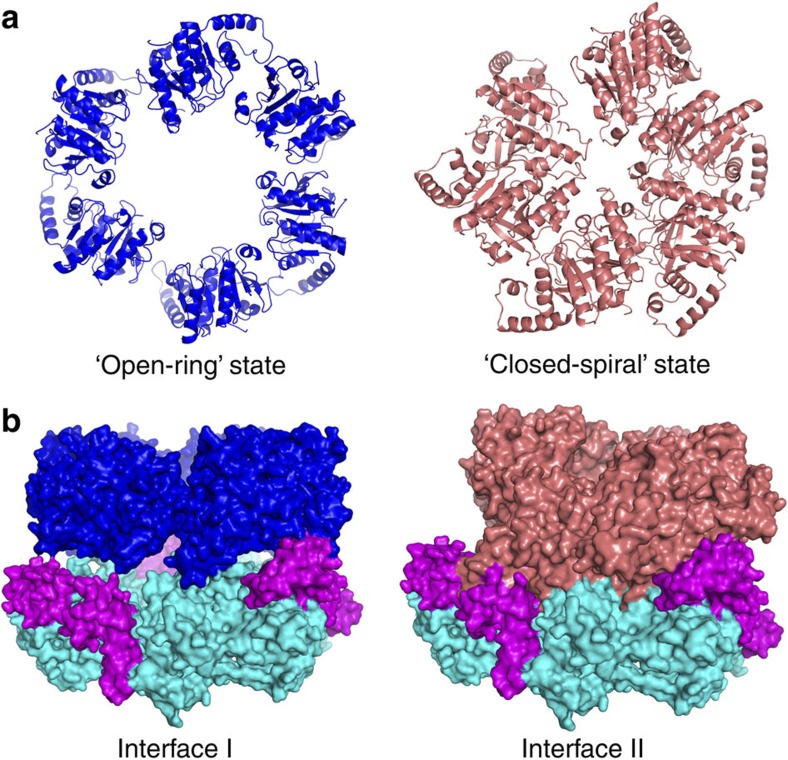
The rings of the CTDs of the helicase and views of the interfaces between the CTDs of the helicase ring and the helicase loader ring. (**a**) The blue and salmon CTD rings of the helicases are from the structure of apo helicase bound to HBD^18^ and the structure of BstDnaB in complex with ssDNA^24^, respectively. (**b**) Two different interfaces are displayed. The colour scheme for BsuDnaI is same as that used in Fig. 3. Left: the interface is from the 570-kDa complex structure. The CTDs of BstDnaB are labelled with blue. Right: the interface between the salmon CTDs of BstDnaB and the BsuDnaI obtained by superimposing the new ‘closed-spiral’ orientations of the CTDs of BstDnaB into the 570-kDa complex is represented.

Our recent structure of BstDnaB helicase in complex with GDP and ssDNA demonstrated a distinct non-planar conformation of the helicase ring representing the translocation state of the ring^24^. The CTDs of BstDnaB helicase in that structure display a new ‘spiral’ conformation, which results in a smaller central channel and tighter packing of the ring (Fig. 5a). Interestingly, when the new ‘spiral’ orientations of the CTDs of BstDnaB are superposed into the 570-kDa complex, obvious clashes between the helicase and the loader were observed (Fig. 5b). Subsequently, a new cryo-EM structure of the *E. coli* DnaB in complex with DnaC, but without ssDNA, demonstrated a different spiral conformation of the helicase and loader. As this spiral conformation has obvious cleft in the two hexamers that could allow ssDNA binding, it is named the ‘open-spiral’ state, and the former spiral is called the ‘closed-spiral’ state. These observations suggest that the binding of substrates, including DNA, induces the conformational changes of the helicase ring and may lead to the loader release.

### Effects of ATP or/and ssDNA on the prepriming complex

**Figure 6:**
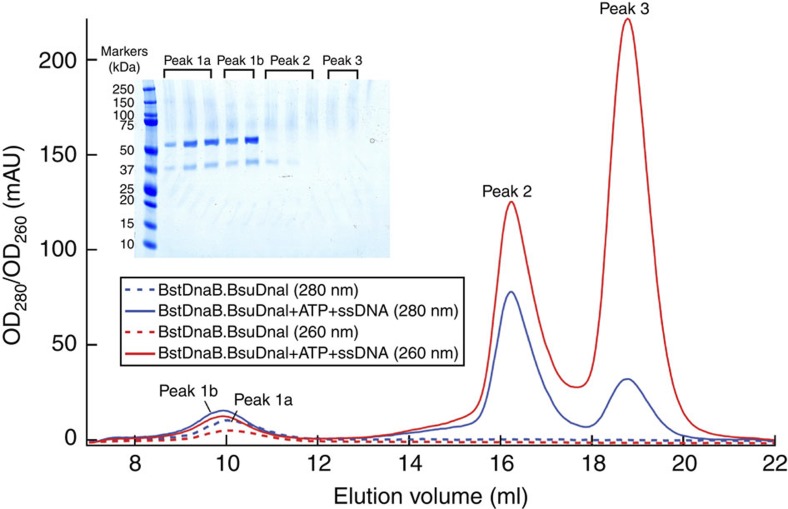
Effects of ATP and ssDNA on the BstDnaB–BsuDnaI complex. The blue and red dash traces represent the migrations of the complex (40 μl, 7.5 mg ml^−1^) on a Superdex 200 10/300 GL column with a flow rate of 0.5 ml min^−1^ at 280 nm and 260 nm, respectively. The migrations of a mixture of the same amount of the complex with 1 mM ATP and three times molar ratios of ssDNA (polyT14) without incubation are demonstrated with blue and red traces (280 nm and 260 nm, respectively). The SDS–PAGE analysis shows the proteins present in the pooled peaks. Combined with other controls and results (see Methods), it is presumed that peak 1a is the DnaB–DnaI complex, peak 1b is the complex together with ATP and ssDNA, peak 2 is the protein BsuDnaI in complex with ATP and ssDNA and peak 3 is the ssDNA and ATP.

To test this hypothesis for the mechanism of the loader release, we utilized size-exclusion chromatography to investigate the effects of these substrates on the prepurified DnaB–DnaI complex. We observed that mixing the complex with ATP and ssDNA (polyT14) resulted in the release of the loader from the complex (Fig. 6). Furthermore, addition of only ATP did not release the loader, but mixing the complex with ssDNA alone led to the appearance of the loader peak (Supplementary Fig. S2b). The presence of the Mg^2+^ did not affect the results of the experiments either. These data suggested that BsuDnaI could load BstDnaB onto the ssDNA in the absence of ATP, which is consistent with the previous report^21^; ATP binding or hydrolysis might not be required for the loader release at this stage. In addition, pre-incubating the mixture at 37 °C for 30 min did not change the results, which indicated the rapidity of the reaction. Replacing the polyT14 ssDNA with a polyT8 ssDNA gave a similar result. Through analysing relative intensities of the bands on the SDS–polyacrylamide gel electrophoresis (SDS–PAGE) gels, it was found that the released BsuDnaI in the peak 2 of Fig. 6 is around 20% of the all loader proteins.

## Discussion

Wickner and Hurwitz^38^ showed that the *E. coli* DnaB and DnaC proteins could form a complex in the presence of ATP. It was then proposed that DnaC is released from the complex via a mechanism that is dependent on ATP hydrolysis^39^. Subsequently, the *E. coli* helicase loader DnaC was proven to be a dual ATP/ADP switch protein^21^. It can bind to ATP, but cannot hydrolyse ATP in absence of the helicase DnaB or ssDNA. The DnaC-ATP state promotes assembly of the helicase DnaB onto the *ori*C and inhibits the unwinding activity of DnaB, whereas the DnaC-ADP state relieves the inhibition of the helicase. Therefore, it was believed that the hydrolysis of the ATP promotes the dissociation of the helicase loader DnaC from the complex and thereby activates the hexameric helicase.

However, recent studies have led to an alternative proposal for when the helicase loader is released from the complex. Soultanas^15^ isolated a stable complex containing BstDnaB, BsuDnaI and BstDnaG by using size-exclusion chromatography and found that this triple protein complex has a poor unwinding activity, but has a good ATPase activity. We purified the 570-kDa complex for our experiments using similar procedures. Galletto *et al.*^37^ utilized fluorescein-labelled *E. coli* DnaB helicase and a statistical thermodynamic model to observe that the nucleotide-binding sites on the DnaC loaders are not involved in the stabilization of the complex; and hydrolysis of NTP bound to DnaB or DnaC is not required for the release of DnaC from the DnaB–DnaC complex. Moreover, ssDNA does not affect the binding of DnaC to DnaB. Furthermore, it was observed that in the presence of the loader, the helicase–primase complex is capable of synthesizing short RNA primers^14^,^31^, which suggested the possibility that the complex of helicase, helicase loader and primase synthesizes RNA primers. More recently, Makowska-Grzyska and Kaguni^35^ found that the hydrolysis of ATP by *E. coli* DnaB or DnaC is insufficient for the release of DnaC from a prepriming complex, which was obtained through first incubating prepriming proteins and DNA, and then purifying the complex by gel filtration chromatography. However, addition of both primase and ribonucleotides results in the dissociation of DnaC from the prepriming complex. Therefore, it was suggested that the release of DnaC is coupled with the translocation of DnaB and RNA primer synthesis, rather than resulting from the hydrolysis of ATP and binding of primase to the complex.

Although the two proposals presented above have been supported by different biochemical data, our structure of the complex and the ‘spiral’ states of the helicase ring in complex with nucleotide and ssDNA^24^ and the *E. coli* DnaB in complex with DnaC suggest an alternative perspective on the release of the helicase loader. Our results lead us to suggest that the helicase ring may undergo the transformations from the ‘open-ring’ state to the ‘open-spiral’ state and then the ‘closed-spiral’ state during the process of the complex binding to the o*ri*C, resulting in the loader ring dissociating from the complex. This hypothesis was tested and was supported by our experiments, which examined the effects of ATP or/and ssDNA on releasing the helicase loader from the prepriming complex. The data clearly demonstrated that *in vitro*, the binding of the ssDNA, not ATP, to the complex leads to a partial release of the helicase loader. Interestingly, Ioannou *et al.*^16^ used surface plasmon resonance experiments to observe that the loader BsuDnaI dissociates from the BstDnaB–BsuDnaI complex after the helicase is loaded onto ssDNA, which also supports our proposal for the release of the helicase loader.

On the basis of our biochemical data, the structure of the complex of BstDnaB, BsuDnaI and the HBD of BstDnaG, the new proposed model, the previously reported data^35^ and the recent cryo-EM structures of *E. coli* DnaB in complex with DnaC^4^,^5^, we can speculate how the loaders are released during the DnaA-dependent primosome assembly.

The helicase–helicase loader complex exhibits at least two different quaternary structures in solution. These two different conformations include a spiral helicase in complex with spiral helicase loader^5^, and the open-ring helicase bound with the open-ring helicase loader^4^. The former is probably the preferred conformation that allows helicase loading onto ssDNA. Binding of the complex with this conformation to the *ori*C would facilitate the transformation from the ‘open-ring’ form to the ‘open-spiral’ form, and also result in significant changes in the relative orientations of the CTDs of the helicase ring as discussed above (from the ‘open-spiral’ to the ‘closed-spiral’ state). These changes will alter the contacts between the helicase and the helicase loader, and presumably facilitate the disassembly of the helicase loader ring, leading to partial dissociation of the loader proteins. This partial release of the helicase loader was observed from our biochemical data (Fig. 6 and Supplementary Fig. S2b). The primase can then be recruited to form an intermediate ternary complex together with the helicase ring and the helicase loader. This ternary complex was also observed from previous studies^15^,^31^,^35^. The incorporation of NTPs by the new ternary complex creates the RNA primers to be used for the elongation stage of the DNA replication. On the basis of the previous report^35^, we presume that with the translocation of the complex along the ssDNA and RNA primer synthesis, the remaining loader proteins in the prepriming complex would be released, which would result in the formation of the functional primosome.

Although the structure of this prepriming complex was derived from a low-resolution map, information that is available from the structure, including interactions, stoichiometry and binding models, is an important supplement to elucidate the mechanism of bacterial primosome assembly, which also has broad implications for the prokaryotic and even eukaryotic DNA replication.

## Methods

### Protein expression and purification

*B. stearothermophilus* DnaB and HBD of primase DnaG were expressed and purified as described previously^18^. The helicase loader protein DnaI fragment was cloned from *B. subtilis* 168 genomic DNA (American Type Culture Collection) using PCR with primers (Integrated DNA Technologies): forward 5′-gcgaccatggaaccaatcggccgttcc-3′ and reverse 5′-gcgactcgagtggatgtcggcggttttctc-3′, and inserted into the *Nco*I–*Xho*I site of plasmid pET21d(+) (Novagen) and expressed in B834(DE3) (Novagen). The cells growing in Luria broth media with 100 μg ml^−1^ Ampicillin (Sigma) were induced with 0.1 mM Isopropyl-β-D-thiogalactoside (Sigma) when the OD_600_ reached 0.6 at 37 °C, and then continued to grow overnight at 20 °C. The collected cell paste was quickly frozen in liquid nitrogen, resuspended in buffer A (50 mM TRIS pH 8.0, 0.3 M sodium chloride, supplemented with EDTA-free protease inhibitor cocktail tablets (Roche)) and lysed using a microfluidizer. The supernatant of the lysed cell paste solution was filtered with a 0.22-μM filter unit (Millipore) and loaded onto a 10-ml Talon superflow metal affinity resin (Clontech) mounted in a 5.0 × 20 cm^2^ Econo column (Bio-rad). After being washed with buffer A using a volume that was 20 times the volume of the resin, the resin was eluted with buffer B (50 mM TRIS pH 8.0, 0.3 M sodium chloride, 0.25 M imidazole). The eluate was then loaded onto a 16/60 or 26/60 Superdex G200 prep grade gel filtration column (GE Healthcare) equilibrated in buffer C (20 mM TRIS pH 8.0, 50 mM sodium chloride). The pooled fractions were concentrated to ~20–30 mg ml^−1^ for the further complex formation. The final purity of the BsuDnaI sample was over 95% as judged by SDS–PAGE analysis and Coomassie blue staining.

### Isolation of the prepriming complex

First, the *B. stearothermophilus* helicase DnaB was mixed with an excess of *B. subtilis* loader DnaI and directly loaded onto a 16/60 or 26/60 Superdex G200 prep grade gel filtration column (GE Healthcare) with buffer C (Fig. 1a). The fractions containing the DnaB–DnaI complex were pooled and concentrated to around 10–20 mg ml^−1^. Extra *B. stearothermophilus* HBD of primase DnaG was then added into this complex and loaded onto a 26/60 Superdex G200 prep grade gel filtration column (GE Healthcare) in buffer C (Fig. 1b). The fractions containing the complex of DnaB, DnaI and HBD of DnaG were pooled, concentrated to ~10 mg ml^−1^ and flash frozen in liquid nitrogen. The final protein complex was over 99% pure as determined by SDS–PAGE analysis and Coomassie blue staining.

### Effects of ATP or/and ssDNA on the prepriming complex

The pure DnaB–DnaI complex (40–50 μl, 7.5–12 mg ml^−1^) was mixed with 1 mM ATP (Sigma) or/and three times molar ratios of ssDNA (polyT14 or polyT8, Integrated DNA Technologies), and loaded onto a Superdex 200 10/300 GL column (GE Healthcare) with or without pre-incubation at a flow rate of 0.5 or 1 ml min^−1^ in buffer C. The ATP and ssDNA were predissolved in 10 mM Tris, pH 8.0. The DnaB–DnaI complex, ATP or ssDNA was also loaded individually onto the column as the negative controls. The reactions were performed in the presence or absence of 1 mM MgCl_2_ as well. The pooled peaks were examined using SDS–PAGE analysis and SimplyBlue SafeStain (Invitrogen). Two wavelengths, 280 nm for measuring protein and 260 nm for monitoring DNA, were utilized in the experiments. The relative intensities of the bands on the SDS–PAGE gels were investigated using KODAK 1D scientific imaging systems.

### Crystallization and data processing

Crystals of the prepriming complex were grown using the condition G7 of the PEGs suite (Qiagen) and the sitting drop vapour diffusion method. Subsequently, the hanging drop vapour diffusion method and microseeding techniques were utilized to optimize the crystals. The optimized condition used was 11–13% (w/v) polyethylene glycol 3,350, 0.2 M lithium sulphate and 50 mM TRIS, pH 8.0–8.3. The complex in the crystal was confirmed by trypsin digestion and protein identification using mass spectrometry (WM Keck Facility at the Yale University). In brief, five to six crystals were washed three times in buffer D (20% (w/v) polyethylene glycol 3,350, 0.2 M lithium sulphate and 50 mM TRIS, pH 8.2), dissolved in double-distilled water and then sent for protein identification. Before flash freezing in liquid nitrogen, crystals were transferred stepwise into a cryo-solution of 30% (w/v) polyethylene glycol 3,350, 0.2 M lithium sulphate and 50 mM TRIS, pH 8.2. Data were collected at beamline 24-ID at the Advance Photon Source and at beamline X-29/X-25 at the National Synchrotron Light Source of Brookhaven National Laboratory. All data were integrated and scaled using the HKL2000 suite of programmes^40^.

### Molecular docking and model building

The C-terminal ring of the hexameric helicase^18^ (2R6C, PDB accession code) and the CTD ‘dimer’ of the loader DnaI^36^ (2W58, PDB accession code) were used separately as search models to obtain an approximate fit of the complex into the cryo-EM density map of *E. coli* DnaB_6_–DnaC_6_ complex^4^ (EMD-1017, EMDB accession code) using MolRep. The fit of the coordinates from the pdb files into the density map by MolRep^41^ was then optimized using the Chimera package^42^ to generate a model containing the CTDs of both hexameric helicase DnaB and hexameric loader DnaI. The N-terminal ring of hexameric helicase and the HBD of primase DnaG were added onto the CTDs of BstDnaB that had been fitted into the cryo-EM map using the coordinates of the 2R6C structure and the programme COOT^43^.

### Structure determination and refinement

The structure was determined by molecular replacement of the model fitted to the cryo-EM map using PHASER^44^. Before being used in PHASER, the B-factors of the model coordinates that had been generated by docking them into the cryo-EM density were set to 20. POINTLESS^45^ was utilized to check the potential Laue groups. The coordinates obtained from PHASER were used to calculate four separate operators, which are responsible for the HBDs of DnaG, the NTDs of the helicase ring, the CTDs of the helicase ring and the CTDs of the loader DnaI ring, respectively, using SUPERPOSE^46^. CCP4 programmes NCSMASK^47^ and DM^48^ were then utilized to generate corresponding masks and averaged maps. These maps were used to manually adjust the positions of the α-helices of the model using COOT. Local real-space refinement in COOT was also performed in the process. The structure was refined initially using cycles of rigid-body refinement and followed by few cycles of restrained refinement using REFMAC 5.0 (ref. 49). The sequence of the CTD of DnaI in the model was changed to that of BsuDnaI using COOT during the process of refinements, based on the analysis of sequence alignment and secondary structure prediction. At this stage, six NTDs of the helicase loader proteins were manually built by positioning the domain structure determined previously by NMR (2K7R, PDB accession code) into 2*F*_o_–*F*_c_ maps and averaged maps using COOT. The density maps in the regions of DnaI and the CTDs of DnaB appear to be poorer compared with those in the other parts of the complex, which might be due to the higher content of β-sheets. The data set was collected, processed and refined at 6 Å resolution. However, the outer shell <*I*/σ*I*> value is 2.0 at 7.5 Å. All figures were created using PyMOL^50^. The real-space correlation coefficients for the fit of each of five domains to both 2*F*_o_–*F*_c_ and omit maps were calculated using OVERLAPMAP^51^.

### Contact analysis and contact area calculation

The intermolecular and intersubunit contacts were analysed using the CCP4 programme CONTACT^47^ with a maximum distance of 5 Å. The calculations of the contact areas are performed using AREAIMOL^52^ with a probe sphere radius of 1.4 Å.

## Author contributions

B.L. performed experiments and solved the structure. W.K.E. performed the multiple-angle light-scattering analysis. B.L. and T.A.S. wrote the manuscript.

## Additional information

**Accession code:** The diffraction data and coordinates have been deposited in the Protein Data Bank under accession code 4M4W.

**How to cite this article:** Liu, B. *et al.* Structure of a helicase–helicase loader complex reveals insights into the mechanism of bacterial primosome assembly. *Nat. Commun.* 4:2495 doi: 10.1038/ncomms3495 (2013).

## Supplementary Material

Supplementary Information Supplementary Figures S1-S2 and Supplementary Tables S1-S2
