# Supplementary material for: Structure of a helicase–helicase loader complex reveals insights into the mechanism of bacterial primosome assembly
Source: Nat Commun. 2013 Sep 19;4:2495. doi: 10.1038/ncomms3495 (PMC3791466; doi:10.1038/ncomms3495)
Supplement: Supplementary Information — Supplementary Figures S1-S2 and Supplementary Tables S1-S2 [file ncomms3495-s1.pdf]

Supplementary information

Structure of a helicase-helicase loader complex reveals insights into the mechanism of bacterial primosome assembly

Bin Liu, William K. Eliason, Thomas A. Steitz*

*To whom correspondence should be addressed. E-mail: thomas.steitz@yale.edu

a

b

c

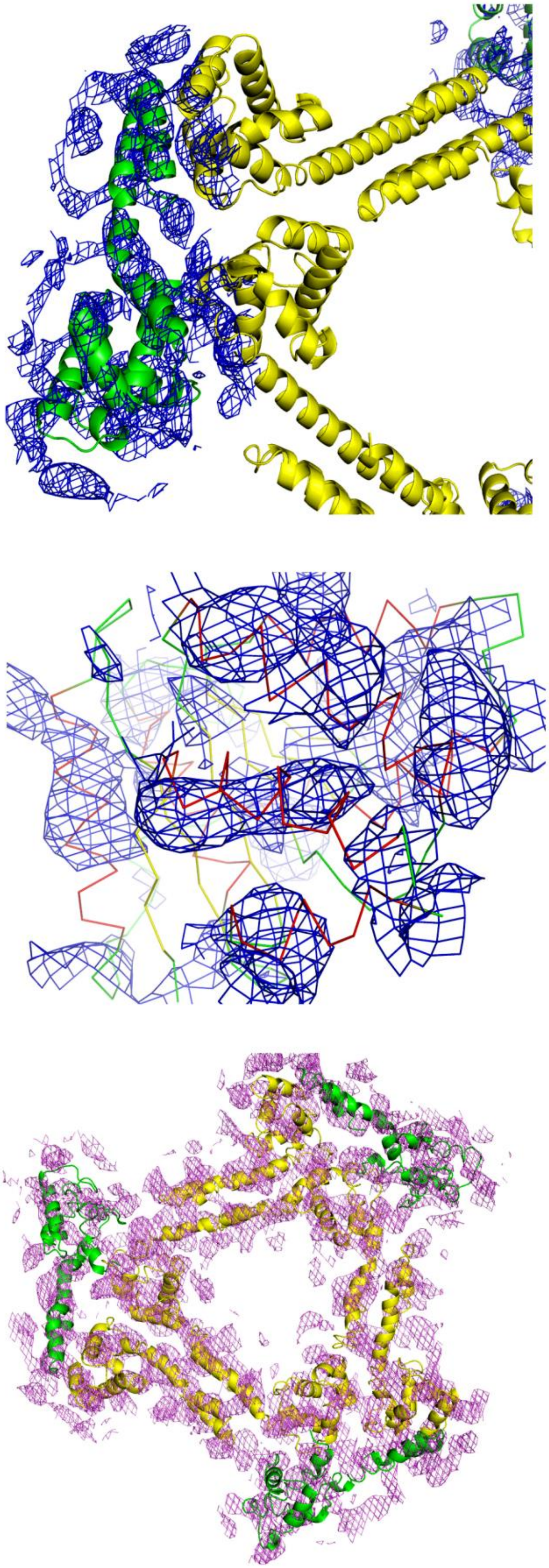

Supplementary Figure S1 | Views of the HBDs of BstDnaG with the NTDs of BstDnaB (a and c) along the *c* axis of crystal lattice and one CTD domain of the helicase loader ring (b). The color scheme in A and C for the domains is same as in Fig. 3. **(a)** The blue averaged map (1 σ), which is superimposed on one HBD, was generated by refining and averaging the BstDnaB helicase without including the HBDs from the beginning. **(b)** One CTD domain of the loader (chain O) is shown with ribbons. α-helices, β-sheets, and loops are labeled with red, yellow and green. Only the portion of the averaged map covering the α-helices is presented, which is blue and contoured at 1 σ, and was obtained through refining and averaging BstDnaB alone without including the helicase loaders from the beginning. **(c)** The pink $2F_o$-F_c omit map, which is contoured at 1 σ, was created by refining the structure after omitting the HBDs of BstDnaG and the NTDs of BstDnaB.

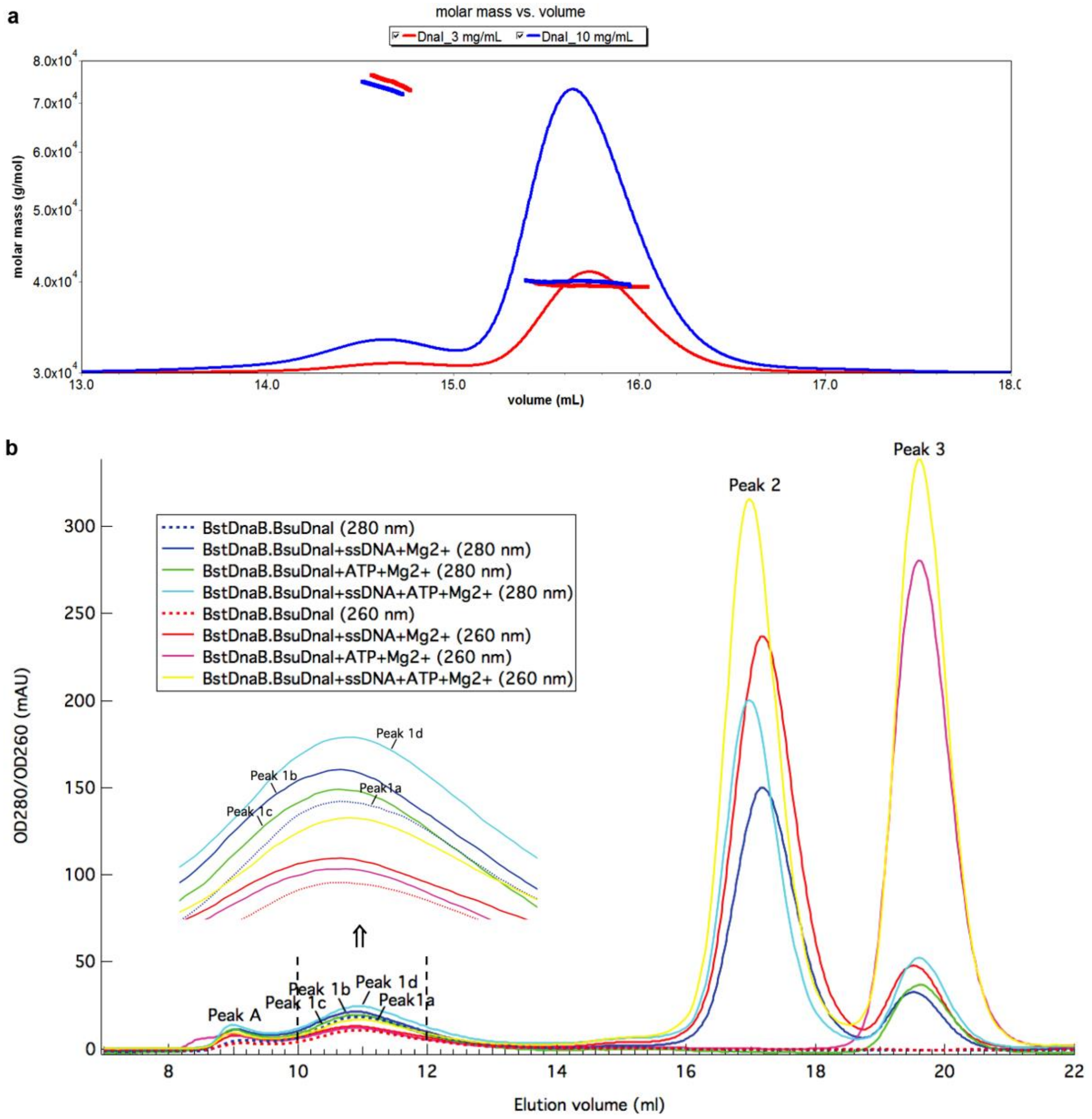

Supplementary Figure S2 | Multiple-angle light scattering analysis of the BsuDnaI protein and effects of ATP or/and ssDNA on the BstDnaB-BsuDnaI complex. (a) Multiple-angle light scattering analysis of the BsuDnaI protein is presented. 100 μL 3 or 10 mg/mL pre-purified BsuDnaI was loaded onto a Superdex 200 10/300 GL column connected by a DAWN HELEOS light scattering instrument (Wyatt Technology Corp.) with a flow rate of 0.5 mL/min using the buffer: 20 mM Tris pH 8 and 0.1 M NaCl. The molecular weights determined from light scattering were ~ 39 and ~ 74 kDa, respectively,

close to the expected molecular weights of a BsuDnaI monomer and dimer. The monomer portion is around 93 % of the all. **(b)** Effects of ATP or/and ssDNA on the BstDnaB-BsuDnaI complex are shown. The experiments were performed using 50 μL 12 mg/mL of the complex on a Superdex 200 10/300 GL column with a flow rate of 1 mL/min. The peaks A in the figure are the aggregate of the complex. The blue and red dash traces, blue and red line traces, green and magenta line traces, and cyan and yellow line traces represent the migrations of the complex, the complex with three times molar ratios of ssDNA (polyT14) and 1 mM $MgCl_2$, the complex with 1 mM ATP and 1 mM $MgCl_2$, the complex together with 1mM ATP, ssDNA (polyT14, 3× molar ratios) and 1 mM $MgCl_2$ at 280 nm and 260 nm, respectively. The peaks 1a, 1b, 1c, and 1d are the complex, the complex with ssDNA, the complex with ATP/ADP, the complex together with ssDNA and ATP/ADP, respectively. Peak 2 represents the protein BsuDnaI in complex with ssDNA or complexed with ssDNA and ATP/ADP. Peaks 3 in the figure individually represent the free ssDNA, ATP/ADP, and the mixture of ssDNA and ATP/ADP. It is clear that no peak could be observed at the position of peak 2 when mixing the complex only with ATP and Mg^{2+}.

Supplementary Table S1 | Data collection and refinement statistics

**Data collection**	
Space group	$P3_121$
Cell dimensions	
a, *b*, *c* (Å)	229.055, 229.055, 364.294
α, β, γ (°)	90, 90, 120
Resolution (Å)[a]	50-6.0 (6.21-6.00)
R_{sym} (%)[a,b]	17.5 (> 100)
<*I*/σ*I*>[a,c]	3.7 (0.5)
Completeness (%)[a]	95.3 (90.7)
Redundancy[a]	3.1 (2.5)
**Refinement**	
Resolution (Å)	20-6.1
No. reflections	17944
R_{factor}/R_{free} (%)	37.91/39.15
No. atoms	31957
B-factors	140.28
Rmsd bond (Å)	0.008
Rmsd angle (°)	1.390

[a] The highest resolution shell is shown in parenthesis. [b] $R_{sym} = \Sigma|I - \langle I\rangle|/\Sigma I$, where I is the observed intensity and <I> is the averaged intensity of multiple observation of symmetry-related reflections. [c] The <*I*/σ*I*> value in the outer shell is approximate to 2 at 7.5 Å.

Supplementary Table S2 | Lists of the real-space correlation coefficients (RSCC*).

Regions	Main chain RSCC_2fofc	Main chain RSCC_omit
HBD	0.6922	0.6707
NTD_DnaB	0.6928	0.6334
CTD_DnaB	0.6879	0.5994
NTD_DnaI	0.6914	0.6627
CTD_DnaI	0.6920	0.6270
Whole model	0.6902	—

* The RSCC values for the fit of different regions to the refined $2F_o$-F_c and omit maps were calculated using OVERLAPMAP.
